# *“That’s your patient. There’s your ventilator”:* exploring induction to work experiences in a group of non-UK EEA trained anaesthetists in a London hospital: a qualitative study

**DOI:** 10.1186/s12909-015-0331-4

**Published:** 2015-03-17

**Authors:** Huon Snelgrove, Yuriy Kuybida, Mark Fleet, Greg McAnulty

**Affiliations:** 1 Education Training and Development, St Georges’s Healthcare NHS Trust, Blackshaw Road, Tooting London, SW17 0QT UK; 2 Clinical fellow in Anaesthesia, Frimley Park Hospital NHS Foundation Trust, Frimley Camberley Surrey, UK; 3 Anaesthetic Registrar, St George’s Healthcare NHS Trust, Blackshaw Road, SW17 0QT London, UK; 4 Consultant in Intensive Care and Anaesthesia, St George’s Healthcare NHS Trust, Blackshaw Road, SW17 0QT Tooting London, UK

**Keywords:** Continuing education, International medical graduates, Anaesthetists, Critical care, Induction to work, Acclimatisation, Safety orientation, Practice-based learning, Socio-cultural theory

## Abstract

**Background:**

European health systems depend increasingly on the services of health professionals who obtained their primary medical qualification from other countries. There has been a significant increase recently in fully qualified specialist doctors arriving from the European Union to provide short term or longer-term solutions to health human resources needs in the UK National Health System. These doctors often take up senior consultant positions. As a result, the NHS has had to learn to deal with both expatriation and repatriation of EU doctors as a constant dynamic characteristic of its own ability to deliver services. We conducted a qualitative study to explore the acclimatisation experience of EU doctors with qualifications in anaesthesia arriving in the United Kingdom to take up clinical employment in the NHS. The question we ask is: how do specialty registered anaesthetists who trained in other European countries experience the process of acclimatisation to practice in the United Kingdom in a large hospital in London?

**Methods:**

We did individual interviews with non-UK, EU-qualified doctors with Certification of Completion of specialty Training who were registered with the General Medical Council in the UK and could practice in the NHS as specialist anaesthetists. The doctors were all interviewed whilst working in a large NHS teaching hospital in London, UK. We analysed qualitative data from interview transcripts to identity themes and patterns regarding senior doctor’s acclimatisation to the British system.

**Results:**

Acclimatisation conceived of as transfer of clinical expertise was problematic for doctors who felt they lacked the right kind of support. Doctors sought different opportunities to share wider perspectives on care deriving from their previous experience.

**Conclusions:**

Hospital conceptions of acclimatisation as a highly individual process can offer an idealized view of clinical work and learning in the new system. Socio-cultural theories suggest we create regular learning opportunities for international staff to critically reflect on practice with local staff to acclimatise more effectively.

**Electronic supplementary material:**

The online version of this article (doi:10.1186/s12909-015-0331-4) contains supplementary material, which is available to authorized users.

## Background

European health systems depend increasingly on the services of health professionals who obtained their primary medical qualification from other countries. In the United Kingdom (UK) overseas trained doctors form nearly 37% of the workforce of which 10% are from the European Union (EU) alone (Table 1).

**Table 1:** **Geographical region of primary medical qualification for UK registered doctors** (**GMC**, **2013**)

PMQ World Region	No. of doctors	%	No. of GPs	%	No. of Specialists	%
EEA11 [1] (excluding UK)	25,503	10.0	3,873	6.30	11,033	15.2
International (excluding EEA)	67,125	27.2	9,999	16.4	17,006	23.4
UK	159,898	63.0	47,199	77.3	44,643	61.4
**Total**	**246**,**064**	**100**	**61**,**072**	**100**	**72**,**682**	**100**

The reasons for this in the UK have been driven by factors including reduced trainee numbers, the European Working-time Directive and changes in demographics, expectations and patient flow [1,2]. The combination of staff shortages in the UK and high labour mobility have also led to increased flexibility of work practices in the National Health System (NHS) in recent years (e.g. the use of employment banks, short term ‘locum’ consultancies, interim fellowships and honorary clinical contracts). These factors, together with tightening of visa conditions for non EU health workers have led to increased numbers of fully qualified specialist doctors arriving from the European Union to provide short term or longer term solutions to health human resources needs. Specialist doctors from the European Economic Area (EEA)^a^ with a Certificate of Completion of Training (CCT)^b^ increased by 13% between 2007–2012 [3]. As a result, the NHS has had to learn to deal with both expatriation and repatriation of EU doctors as a constant dynamic characteristic of its own ability to deliver services. Among the ramifications of this has been a need to cope constantly with ‘induction’ to work of new staff, but also exposure to a rich diversity of employees who are often highly knowledgeable and experienced in their understandings of different European health systems. Surprisingly, despite the rising numbers of EU specialist hospital doctors arriving into the NHS, their retention, morale and educational needs have not been strongly focussed upon [4,5] and the cross cultural adjustment of ‘elite’ migrant doctors is, in particular, under researched in the scholarship and policy terrain on acclimatisation [6].

The kinds of challenges doctors face on arriving in hospital systems in Anglophone countries have been reported in the literature in small studies from Canada, USA, Australia and New Zealand [7-14]. These have focussed principally on induction of International Medical Graduates (IMGs) coming into postgraduate training programs in a different culture and refer mainly to primary care settings. The word ‘induction’ in the literature (from Latin *inducer*: *to lead to*) has metaphoric complexity and embraces a view of learning comprising notions of ‘acculturation’, ‘orientation’ ‘acclimatization’, ‘adaptation’, ‘compliance’ and ‘standardisation’ to name a few. We use the word ‘acclimatisation’ to encompass this complexity while acknowledging it refers to a period of dynamic learning which is extended over a lengthier period of time than the use of ‘induction’.

Most of the studies describe subjective personal experiences of the transition to a new system with its corollary of general cultural linguistic and medical-cultural challenges. These include feelings of professional inadequacy including a lack of knowledge and being deskilled [7-10], losing status and weakening professional identities, being isolated, feeling culturally unaware [10-12,14] and being linguistically challenged [7,8,10,13]. In these studies there is an underlying emphasis on pre-ordained competencies and professional rather than organisational socialisation where the focus on learning in the new environment is often subsumed in concepts of transfer and *up*-*skilling* or *re*-*skilling* through some sort of pre-eservice induction training [15]. This problem-oriented view of the ‘new arrival’ tends to privilege public regimes of justification [9]. Some recent studies have embraced instead a more extended idea of induction of health care professionals as a process of acclimatisation and learning through ‘regimes of familiarity’ [16] ‘critical reflective processes’ [14], the ‘enterprising learner’ [17] and notions of growing ‘entrustability’ [18] all of which draw on situated and social process models of adaptation and learning in new environments. These have in common a call for comprehensive and expanded organisational support over longer periods of time [14,18,19].

Interestingly, in the expatriate literature on elite professional migration experiences in fields outside of healthcare, similar challenges related to migration from one learning environment to another are reported [20-22]. However, here there is a stronger positive emphasis on what more senior international arrivals *bring* to the new system. Common themes in this literature concerning acclimatisation point to the need for opportunities for local and foreign professionals to engage not only in discussions about the challenges experienced or cross-cultural awareness raising, but also, and crucially, dynamic negotiations of their leadership roles and of ways they can contribute to improve local practices. This appears to be far less emphasised in the healthcare literature.

The question this paper asks is how do specialty registered doctors who trained in other European countries experience the process of acclimatisation to clinical practice in the United Kingdom in a large London hospital? The paper will draw on socio-cultural theories of learning to try to glean insights into how to support new fully qualified international staff more effectively.

## Methods

### Participants

Eight non-UK EU-qualified doctors with CCT certification working in a large teaching hospital in London volunteered to participate in the study between May 2013 and February 2014. Inclusion criteria were that doctors were registered with the GMC and could practice in the NHS as specialist anaesthetists. Ages of the volunteers ranged from 33–46 years. While all participants were eligible to apply for consultancy positions, three preferred to take on more junior clinical roles. The period elapsed since arrival in the NHS spanned from 8 years to 18 months. Table 2 indicates the EU countries of origin and the dates of participants’ primary medical qualification (PMQ).

**Table 2:** **Country of primary medical qualification**

Grade	Sex	Country of primary qualification	PMQ	Specialty Registration In UK
Clinical fellow	F	Italy	2006	2012
Consultant	F	Italy	1989	2003
Consultant	F	Germany	1991	2004
Clinical Fellow	M	Greece	2001	2011
Consultant	F	Greece	1998	2005
Staff grade dr.	M	Poland	1996	2002
Consultant	M	Germany	1990	2009
Clinical Fellow	M	Spain	2007	2012

### Data collection

Anaesthetists were interviewed individually for 60-minutes by H.S. Recruitment was opportunistic and performed by personal contact by two co-researchers: a consultant anaesthetist (GM) and an anaesthetist registrar (YK). Participation was voluntary and written informed consent was received from all interviewees. Ethical approval exemption was given by the National Health Service Ethics Review Board.

Interviews were filmed and audio recorded. The content of the semi-structured interview guide was prepared by informal exchanges with expert informants and by conducting a literature review. The resulting interview schedule was piloted with 2 volunteers from another hospital. The interview guide consisted of open-ended questions designed to encourage free expression and generate responses related to doctors’ personal experiences, in particular the challenges they faced as they started work in the NHS, All interviews were conducted in English. The appendix shows the semi-structured interview questions and prompts (See Additional file 1).

### Data analysis method

The interviews were transcribed verbatim from digital recordings. After transcribing the interviews copies were sent via email to each interviewee to receive respondent validation [23]. Reply emails were included with the interview corpus for detailed analysis. Drawing on grounded theory [24], we initially used independent ‘open coding’ involving attaching conceptual labels to segments of data and then comparing our codes. Although our recruitment of doctors was opportunistic, we did not decide beforehand an upper limit of the number of doctors to interview. Instead, in our thematic analysis we found that saturation, or the point at which no new information or themes are observed in the data was evident after 9 in-depth interviews, and that basic elements for metathemes occurred as early as five interviews. During independent thematic analysis the researchers HS, GM and YK arrived at consensus and clustered codes into discrete categories to develop an organizing structure to illustrate relationships. Through coding categorization and analytical reflection more subtle themes were generated. We then compared and re-examined these looking for similarities and differences across categories.

Three emerging themes were then applied to the full set of individual transcripts through the use of a coding glossary [25]. These are summarised in Table 3.

**Table 3:** **Coding themes**

Themes	Sub categories
**Crossing borders & acclimatisation**	Formal and extended induction process
	Language and communication
**Patterns of participation**	Personal agency, legitimacy and status
**Learning and supporting expertise**	Learning with others

**Figure 1:**
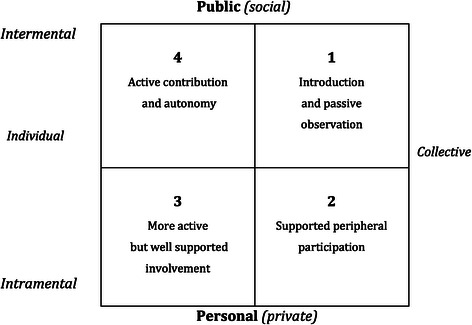
**Harré’s [** 17 **,** 18 **] two-dimensional space based on Vygotsky’s ideas on assisted performance.**

We drew on socio-cultural theories from Vygotsky [26], Harré [27,28] and Edwards [29-31] related to ideas of assisted performance to illustrate how themes and concepts systematically interrelate. The model in Figure 1 assumes that in the process of learning and identity development individuals move from quadrants 1 through 2 and 3 to 4. It shows how individuals in formal and informal environments are able to engage with others in order to approach, assess and respond to the possibilities of action available to them in a situation. The capacity to move to and fro across the quadrants to support learning is what Edward’s calls “relational agency” [30]. While this model was historically used to describe learning by individuals, it has more recently been deployed to account for collective learning in organisations where the emphasis is on the creation of transformative learning, rather than transmission learning [32,33] This shift in approach from individuals, describes how learning emerges in institutionally situated activity and how higher mental functions arise from collective forms of behaviour, and from ‘multivoicedness’, where contradictions and dissonance are thought of as a creative source of change and development [34].

## Results

### 1. Crossing borders and acclimatisation

The combination of moving country, changing language and a new hospital system created a strong psychological sense of uncertainty, particularly among consultant doctors. Interviewees said that they were ill prepared for the realities of clinical practice in the UK and surprised that formal hospital induction was non-specific to foreign doctors, consisting of just 2-days of seminars on occupational health and general hospital procedures. The interviews reveal how doctors therefore looked to extend their induction experientially and informally and how they tried to reconcile discrepancies in their understandings.

### Formal and ‘extended’ induction

One senior consultant described the message she received from the formal induction as “a very objective view of acculturation”.

- I heard these descriptions of routines and rules and stable patterns of activities and protocols [ ] and unfamiliar words to describe them, that the English staff all recognized and understood, and that everyone adopted. So I had to learn them too, to be a consultant here

A recurrent motif across interviews regarding induction is an idea characterized by the objectification and standardization of shared understandings; a ‘demand’ to learn a ‘common language’ to quicken knowledge transfer. For interviewees, however, transfer was more than just common language. A senior consultant described how she sought to “find out what they really expected of me” by shadowing future colleagues in theatres for two full weeks:

- I observed and tried to just, probably got on lots of people’s nerves. But for me it was really reassuring to see how people did things and asking questions and you know [ ] standing on the edge but looking in. It was my way to know the system!

This self-initiated acclimatisation “without pay” was an opportunity for her to constitute and re-constitute her ways of ‘knowing’ in the new organisational culture (See Figure 1). In fact, the capacity to be ‘expert’ was a recurrent concern for all the doctors with senior roles. Most reported feeling that they needed to reconfigure their professional identities downwardly. One doctor said his expertise had ‘shrivelled’ while another remarked:

- I remember for the first four to eight weeks, I felt very uncomfortable on very, very thin ice. Like being considering myself as a very experienced anaesthetist. That I could fall.

The consultant’s perceived capacity to interpret and use the tools in his environment to act clinically was key to his sense of being an expert but the cultural shift meant his expertise was now reconfigured more as a dynamic on-going accomplishment, to stay upright, than a static transferable competency from one country to another.

For the hospital, the CCT credentials indicated this doctor was ready to practice in the UK. However, consultants interviewed said they were expected to contribute activity and autonomously too early on, often before they had developed sufficient social capital to be able to perform at their previous levels of expertise.

- I wasn’t supported terribly, I have to say. I had a couple of quite difficult times during the first months and then after that I requested to have a mentor, a person with whom I could meet. I am like: ‘Sorry, I am completely new to the system, [ ] but induction works for everybody [ ] probably from the UK, who starts in this Trust. So you just learn a couple of things, but all the important things for you coming in from another system you just, at times, have no inkling.

### Language and communication

Interviewees were acutely aware of the effect language skills have, even though all could communicate fluently. The simplest tasks of measuring, answering phones and interpreting abbreviations could cause discomposure. One anaesthetist describes her alarm on reading the patient notes in theatre.

- “Oh, we are having an admission: the AAA^c^ repair”, and I didn’t know what AAA was. And I was afraid to ask what is the AAA repair because everybody reacted around me, this is well known. I open the notes and I will see the scribble, it will not help me. So I was like… The first week I was just treating the monitors, I will look the monitors around me, I was like in the fight or flight mode, yeah, in the middle of the ICU^d^. I looked at the monitors and I was treating the monitors not knowing actually how the patient ended up there.

Amidst this abundance of culturally saturated tools her embarrassment was obvious when the surgeon later rejoined:

- “AAA? Well, it’s not Moody’s rating of the Greek economy!”

Reflecting on this episode she remarked that in Greece the use of acronyms is ‘not legal’ on patient records because they can confuse people. This was a sobering afterthought in a system where written and verbal communication failures are among primary causes of serious incidents in UK hospitals [35].

Although formal mastery of English eclipses our discussions of non-locally trained staff, it is not at issue here. Instead there is a broader conception of communication competency that is more congruent with our understandings of how safety emerges through collective shared understandings. A consultant level doctor who is new to the culture and ‘*in fight or flight mode*’ admits to being reticent. Yet what is crucial for *all* staff in critical care contexts is how reflexively aware they are regarding their understanding of events and *how* they construct, strategically, meanings with others and reconcile discrepancies in practice. This aspect of communication competence cannot be addressed with a pre-emptive reliance on formal language testing alone^e^. Doctors interviewed said we needed to contextualize the learning of language strategies with local staff within existing frameworks commonly used to improve the communications skills of local staff; for example, through extensive opportunities to discuss and practice clinical non-technical skills and through simulation-based team exercises.

### 2. Patterns of participation

Doctors’ accounts of early and effective participation in practice unfolded along axes of ‘autonomy’ and public ‘scrutiny’ revealing their efforts to manifest leadership and expertise. Interviewees agreed that junior clinical fellow posts generally established doctors within a network of supervision and support which facilitated their ability to ‘feel safe’ and ‘acclimatize’. However, they complained of lack of autonomy. In contrast, two consultants provided starker descriptions of feeling under scrutiny and the emotional impact this had on them. A common theme was adjusting to more independence and autonomy; ‘to people’s expectations that you lead’. A German consultant described his initial disorientation:

- It was because of the independence you have here and very often you work on your own and you make your decisions on your own. Let’s say, where I come from, if you work in an anaesthetic department you have a sort of corporate identity in a way how we do things. If you do general anaesthetist, let’s say you do a colorectal list which I did at the beginning, you would have all the anaesthetists in the department, especially the senior ones, would have agreed on how to do this colorectal list so the variation in treatment and the variation in doing things would be minimal.

When expert practice was no longer sustained by a ‘corporate identity’ and consensus, greater autonomy caused an unsettling perception of unconformity. Consultants found they had to convert their clinical knowledge and expertise into localized solutions ‘on a new block’ but that their expertise was now suddenly stripped of its previous contextual richness. An Italian consultant described this predicament:

- I was on my own on the block. And that was my list. That was my ventilator. That was the patient [*waving as if to say goodbye*] ‘Have a good day!’ I was completely left alone to face the cultural differences, the technical differences, nobody would tell me the list works this way or that way.

The need for more opportunities to reconstitute their knowledge in conversations about practice is a recurrent theme in interviewees’ reflections on their learning. In Vygotsky’s ‘intramental’stage (Figure 1–quadrants 2–3) what is valued in a social context is assimilated into how people think. When these understandings are ‘externalised’ in Harré’s [27] (stage four they are enacted ‘publicly’ and reconstituted. This is central to Vygotsky’s [26] vision of human agency. From a socio-cultural perspective, hospital systems that are adept at learning should be ones in which professionals, irrespective of their seniority, find it easy to go to and fro between the areas of support so that they can reconstitute their knowledge and contribute to practice. Consultants in this study lacked local contextual understandings but found it difficult to engage in recursive ‘feedback’ loops in the clinical environment, and this was frustrating.

### Personal agency, legitimacy, status

Interweaved in doctors’ accounts are recurrent episodes which show how they tried to shape a distinctive notion of self–identity, but struggled at times to reconcile this with their social-identities. One new consultant describes how she felt the surgical team wanted to ‘test her professional mettle’ and of the psychological ‘knock-on effect’ this had which lead to a ‘near miss’ incident. Her patient suffered a sudden, potentially dangerous fall in oxygen saturation during anaesthesia and she was unfamiliar with the equipment:

- At the time I felt as if everybody’s eyes were just on me, watching me, how I was doing, what I was doing, and I felt that I couldn’t fail. So before calling for help or calling, you know, this is a risk situation, and again, because of cultural reasons, I was like, may be I should handle this on my own. People will be like “this is a [*pauses*, *opening hands in a gesture indicating hopelessness*] a foreign doctor, and we’re expecting her to have problems, we’re expecting her not to be up for the challenge, for the post, and so it’s like ‘let’s see how she does’.

This is not simple a fragment of autobiographical narrative but also a collective storyline concerning an operating team. Harré’s model is useful here because it shows us how professional identity is mutually constituted and how this construction comprises both individual and social factors. If what one says or does cannot be joined in the locally accepted cluster of behaviours that define the ‘professional persona’ that person is treated with distrust. The anaesthetist’s reflection on this event is an insightful, if belated realization of the need to engage in a different way with her team.

- I was expected to lead. That was the main problem. It took me a long while to understand how much of that was required of me. I had to lead. I had to voice what I wanted. I had to voice my thoughts, what I thought was right or wrong for that situation and I’d prefer to do this instead of keeping all to myself, essentially in Italy that didn’t matter I just had to get on with the job, [*in Italy*] try not to bother the surgeon and just churn operation on operation

To break the cycle of professional isolation she realised she needed to voice her clinical reasoning and decisions to others. But also that local staff needed to recognize motives and resources she brought to bear on the task to align their *own* responses. From the socio-cultural perspective the expectation ‘to lead’ here is to construct shared understanding by working together to interpret the problem as well as to respond to it. It rests on what Edward’s calls ‘relational expertise’ [30].

In another instance of this a consultant challenged the customary ways of doing things that were embedded in the organizational culture:

- Hang on! If there is really something I really don’t want to do, and they’re doing it here, I still don’t play the, you know, play the game, because if I feel it is unsafe. So I felt, you know, I felt I had the ability, although it was obviously stressful, to speak up and say: “Actually, I think this is not how I should do things. I think I might do it this way.

Instead of simply ‘internalizing’ the local knowledge and how it is manifested in a routine she contests it by ‘speaking out’. By refusing to adapt - to ‘play the game’ of how things are routinely done – she *reinterprets* and thereby potentially *reshapes* the practice. This is the meaning of Harré’s ‘public self’ in quadrant 4 – the acquired confidence to contribute something new and to openly voice one’s position.

### 3. Learning and distributing expertise

Doctors interviewed felt that there was a need to harness the ways of seeing that new senior staff bring into the hospital from other systems. And that contested practices were never entirely a matter of personal professional autobiography or membership of a distinctive cultural group, but rather an opportunity to open up dialogic spaces from which both new staff and the system could benefit. One consultant, for example, described his ‘awful uneasiness’ that after delicate anaesthetic procedures patients were sent back to the wards rather than to High Dependency Units (HDU).

- You know, if you do stuff to them that is slightly dangerous, for example, a thoracic epidural, you would want them to be monitored at least in an HDU environment, whereas most of my patients I have to cope with are going back to the ward. And I remember, every Monday, I was calling the ward at 8 o’clock at 9 0’clock in the evening and then again at midnight because I was very worried about what I could do to my patients because I obviously wasn’t used to that. I didn’t trust that the system would work.

Closely tied to the need for more opportunities to share their own wider perspectives on care with colleagues, was another, to have their own professionalism acknowledged. A consultant explained:

- So it’s like “I really don’t understand why you did this” and it’s like “This is why I did that, and it’s a perfectly acceptable reason why I did that. If you’re telling me that I should just do things one way, just say so, but it’s not quite an explanation of why I should do things, that way. It’s not a protocol or a guideline. So at times, it’s like, “can we just talk about cultural differences and that was my attitude at the time “I’m very willing, which is why I requested this mentorship in the first place. I’m very willing to try and understand what’s appropriate for the system because I haven’t come here to establish a new rule; I’ve come here to embrace a new system. But, you know, I’m not a novice, clinically!”

The search for ‘spaces’ where divergent meanings of how to do things could coexist, and extended ideas of clinical understanding emerge, is a distinctive strategy of doctors’ resilience during their acclimatisation. Among ‘spaces’ consultant doctors thought should be early components of induction processes for senior staff were not only the obvious options of having mentors and buddies but also of ‘being assigned early on as mentors to others’, opportunities to ‘teach junior staff’, to contribute knowledge in formal case studies or presentations and opportunities to participate in and contribute to local educational activities.

## Discussion

This small study aimed to explore the experiences of EU non-UK consultant level anaesthetists arriving at our hospital to take up employment from other European countries. It reveals that the process of acclimatisation into the NHS extended over several months and was often a stressful one. It challenged the doctors’ own sense of identity, status and sometimes their value systems. Similar themes have emerged in the wider literature on IMGs as well. However, this study focused on specialty doctors who were working as consultants, or eligible to work as consultants in the UK, and whose challenges were greater because they had heavier clinical responsibilities and greater autonomy.

In the light of NHS employment practices characterised by flexibility and mobility this study foregrounds how newly arrived non locally-trained specialists in our hospital were left largely to acclimatise ‘alone’ to meet service delivery needs. This appears to reveal an understanding of ‘preparedness’ for practice that is based primarily on an idea of individualised ‘credentialisation’ of learning. In other words, it shows a bias towards a pedagogy of individual acquisition where more senior doctors just need to ‘learn on the job’, and an enduring idea of decontextualized and unproblematic transfer of learning from previous experience in other countries [15]. The doctors in this study clearly contested the assumption behind a short formal hospital induction programme for new staff, that this was in any way sufficient to pre-empt the cycles of professional isolation and feelings of unsafeness they said they all experienced in their first several months on the job after arriving from another country.

In contrast, the theoretical insights developed from the Vygotskian tradition by Harré [27,28] and Edwards [30,31] provide an insightful critique of transfer and adaptation approaches because they suggest that human action and development arises through negotiations between the social and the personal worlds in ways that are relational rather than fixed or static. This was borne out in the interviews, in different ways and with different emphases, where ‘induction’ to practice was reconceptualised as an extended interactional process that should enhance knowledge sharing. Consequently, what newly arrived foreign consultants said they needed to break the cycles of professional isolation they had experienced were more formalised opportunities to observe others, to be observed and to engage more systematically in conversations about practice with colleagues. Part of this concerted effort should encourage opportunities for new staff to reformulate problems they encounter and challenge certain aspects of the culture and routine practice in the host country workplace. Interestingly, such challenges were seen as key to learning about how things were done, but also to developing meaningful communication and reciprocal trust with local staff and teams.

Simple and economic strategies to support new arrivals could include regular group meetings with local staff to reflect on everyday practical challenges. Involvement in simulation-based training in the hospital could also provide opportunities for feedback seeking and giving and, importantly, an occasion to raise cross-cultural awareness among local staff. Assigning mentors and buddies to all grades on arrival is also highlighted. These sorts of strategies are not new, but most are usually reserved for more junior doctors and trainees rather than with consultant level doctors working on the shop floor.

Interestingly, what distinguished the needs of more senior doctors in this study was the desire to acclimatise to the hospital in proactive ways, for example by involvement in quality improvement teams and through teaching and mentoring more junior staff. These activities would hone their communication skills and strengthen their system knowledge and relational expertise while preserving their professional identities during the first months of acclimatisation when they reported they felt most exposed.

This study has many limitations. Firstly, it represents a small cross section of specialist practitioners in our hospital and by not following up on interviews we have risked getting only a snap shot of doctors’ views. Although the opportunistic sample of anaesthetists represented a mix of grades, ages and gender, it is likely that some participated because they particularly wanted to share their story. This might have led to the selection of some unduly pessimistic or optimistic views. Importantly, how the doctors developed strategies of resilience in the face of hardships is left largely unexplored, although this is a key to understanding processes of acclimatisation and adaptation. Furthermore, all interviews were done in English, which might have prevented some participants from being able to express the nuances of their experiences fully. A broader range of specialties, and professional roles could also have provided richer insights. Finally, the inclusion of ethnographic observation methods would have strengthened the findings considerably by bringing empirical observations to bear on personal accounts and recollections. Future studies will need to incorporate our knowledge of the complex layering and dimensionality of doctors’ experiences emerging here with new programmes designed to strengthen on-the-job confidence.

## Conclusions

Senior doctors arriving from abroad expect adequate organisational orientation and support over time to acclimatise and integrate into the new system to deliver quality care. We can do more to acknowledge how experienced doctors’ own interpretations of clinical practice and events that comprise their acclimatisation learning experiences provide opportunities to reconstitute knowledge individually and collectively in the system. To harness this experience and support new staff requires a more cohesive institutional approach. Needless to say, support is not a substitute for personal expertise and individual responsibility, but an organisation which opens up dialogic spaces with the knowledgeable rather than the novice newcomer, is surely a more resilient one.

### Endnotes

^a^European Economic Area: includes EU countries excluding Croatia plus Norway, Iceland and Liechtenstein.

^b^The CCT is awarded to specialist doctors under Annex V (point 5.1.2) of Directive 2005/36/EC for the UK. The certificate confirms satisfactory completion of specialist training.

^c^Abdominal Aortic Aneurysm.

^d^Intensive Care Unit.

^e^The NHS made it a requirement on the 18^th^ June 2014, that non-UK EU doctors acquire an International English Language Testing Score of 7.5 (NHS Employers).

## Additional file

Additional file 1: **Interview Schedule.** Semi-structured interview Schedule for EU trained anaesthetists Interviews were approximately 60-minutes long.

## Abbreviations

AAA: Abdominal aortic aneurysm
CCT: Certificate of completion of training
A CCT in: Anaesthetics provides its holder with recognition of this qualification within Europe
EEA: European Economic Area
EU: European Union
HDU: High Dependency Unit
ICU: Intensive Care Unit
IMG: International medical graduates
NHS: National Health Service, United Kingdom
UK: United Kingdom
