# Supplementary material for: “That’s your patient. There’s your ventilator”: exploring induction to work experiences in a group of non-UK EEA trained anaesthetists in a London hospital: a qualitative study
Source: BMC Med Educ. 2015 Mar 17;15:50. doi: 10.1186/s12909-015-0331-4 (PMC4367902; doi:10.1186/s12909-015-0331-4)
Supplement: Additional file 1: — Interview Schedule. Semi-structured interview Schedule for EU trained anaesthetists Interviews were approximately 60-minutes long. [file 12909_2015_331_MOESM1_ESM.docx]

**Semi-structured interview Schedule for EU trained anaesthetists**

Interviews were approximately 60-minutes long

Prompt questions and back up questions for semi structured interviews designed to encourage free expression and generate responses related to doctors’ personal experiences, in particular the challenges they faced as they started work in the NHS,

**1. Motivations and employment process**

Tell us how you ended up in the UK and what motivated you to apply for a position in the NHS

Was the position you applied for equivalent to the one you had back home*?*

**2. Challenges for international staff**

What surprised you when you started work? *(e.g. What differences struck you? Cultural? Organisational?)*

What did you find attractive about working here? What was less appealing?

Can you tell me what was most challenging for you?  *(e.g. communication, language, hospital organisation, roles and responsibilities)*

How did you see your position in the clinical workplace?  *(e.g., your role, type of relationships, status, hierarchy)*

How do you think other people saw you (nurses, peers, consultants?)

What made you feel valued? (or not valued)

How do you think doctors trained abroad have different needs to locally trained new arrivals? (if any)

**3. Induction, acclimatisation and learning**

What formal or informal support did you receive? *(e.g. individuals? documentation? Formal learning opportunities; cultural linguistic socialisation?)*

What did you think of the support?

Clinically speaking, were expectations people had of you different from your previous experiences?

Have you always been comfortable with these expectations?

Can you recall any situation when you felt you were getting out of your depth and needed help? What did you do?

Can you reflect on any critical incidents where you got particular insights into local practices? (e.g. into clinical care, system safety or cultural differences that you needed to appreciate)

From your experience, how could the hospital support new consultant level doctors in future
